# Pandemic Influenza Planning, United States, 1978–2008

**DOI:** 10.3201/eid1906.121478

**Published:** 2013-06

**Authors:** John Iskander, Raymond A. Strikas, Kathleen F. Gensheimer, Nancy J. Cox, Stephen C. Redd

**Affiliations:** Centers for Disease Control and Prevention, Atlanta, Georgia, USA (J. Iskander, R.A. Strikas, N.J. Cox, S.C. Redd);; Public Health Consultant, Cambridge, Massachusetts, USA (K.F. Gensheimer)

**Keywords:** pandemic influenza, influenza, public health preparedness, pandemic planning, viruses

## Abstract

During the past century, 4 influenza pandemics occurred. After the emergence of a novel influenza virus of swine origin in 1976, national, state, and local US public health authorities began planning efforts to respond to future pandemics. Several events have since stimulated progress in public health emergency planning: the 1997 avian influenza A(H5N1) outbreak in Hong Kong, China; the 2001 anthrax attacks in the United States; the 2003 outbreak of severe acute respiratory syndrome; and the 2003 reemergence of influenza A(H5N1) virus infection in humans. We outline the evolution of US pandemic planning since the late 1970s, summarize planning accomplishments, and explain their ongoing importance. The public health community’s response to the 2009 influenza A(H1N1)pdm09 pandemic demonstrated the value of planning and provided insights into improving future plans and response efforts. Preparedness planning will enhance the collective, multilevel response to future public health crises.

## Historical Background

Influenza pandemics occur when an animal influenza virus to which humans have no or limited immunity acquires the ability, through genetic reassortment or mutation, to cause sustained human-to-human transmission leading to community-wide outbreaks (*1*). The existence of a pandemic is currently determined by the extent of disease spread, not by the lethality of the disease caused by the novel virus (*2*). During the twentieth century, influenza pandemics occurred in 1918, 1957, and 1968. The 1918 pandemic, known as the “Spanish flu” pandemic, was unique in that the highest number of deaths was among young, healthy persons. Excess mortality in the United States during the 1918 pandemic was estimated at 546,000 deaths (*3*). The pandemics in 1957 and 1968, although associated with death rates greater than those for seasonal influenza epidemics (*3*), were far less devastating than the 1918 pandemic.

Before 1976, public health planning for pandemics primarily occurred in response to detection of a novel influenza virus. This reactive mode continued despite the framework outlined in 1960 by US Surgeon General L.E. Burney for responding to the next pandemic. That framework involved recognition of the pandemic (i.e., surveillance), manufacture and distribution of vaccine, and identification of research needs (*4*). Large-scale infectious disease response planning may have been hampered by the tacit assumption that the government’s public health resources were better directed to other priorities.

In January 1976, a novel swine-origin influenza virus emerged among soldiers at Fort Dix, New Jersey (*5*); 1 soldier died, and an estimated 230 were infected. The emergence of influenza virus of swine origin at Fort Dix led to the decision to mount a national immunization program (*6*). The following events occurred subsequent to this decision: Congress funded vaccine production and liability indemnification of manufacturers, vaccine was produced, a mass immunization campaign commenced, and 45.65 million persons were vaccinated in the United States (*7*). Initial fears that the virus would cause a pandemic did not materialize: sustained transmission did not occur outside of Fort Dix. The vaccination campaign began in October 1976 and was halted in December because of initial reports of a rare association between the so-called “swine flu” vaccine and Guillain-Barré syndrome; the association was later confirmed (*7*). An influential policy review of the “swine flu affair” (i.e., the campaign to immunize the US population against a possible epidemic) identified several critical needs for future planning: 1) a more cautious approach to interpreting limited data and communicating risk to the public, 2) greater investment in research and preparedness, 3) clearer operational responsibilities within the federal government, 4) clear communication between planners at all levels of government, 5) strengthened local capacity for plan implementation, and 6) improved mechanisms for program evaluation (*8*).

In November 1977, separate from the Fort Dix outbreak, a strain of human influenza A(H1N1) virus reemerged in the former Soviet Union, northeastern China, and Hong Kong, China, even though the virus had not circulated since 1957. This strain primarily affected young persons, and caused mild illness (*9*). The virus was found to be closely related to a 1950 A(H1N1) strain but dissimilar to the 1957 strain, suggesting that this 1977 outbreak strain had been preserved since 1950 (*9*).

The confluence of fears of a possible pandemic in 1976 followed by the reemergence of a new strain of circulating seasonal influenza virus in 1977 led to focused pandemic planning efforts in the United States. The primary purpose of this article is to describe US pandemic planning during 1978–2008, just before the onset of the influenza A(H1N1)pdm09 pandemic in April 2009. We believe that understanding the historical and policy context within which the A(H1N1)pdm09 pandemic occurred is helpful in assessing the implications of pandemic planning for responses to future pandemics and for ongoing infectious disease preparedness efforts.

## Sources

We conducted searches of the medical literature and key websites (e.g., www.pandemicflu.gov) for peer-reviewed manuscripts and published governmental plans relevant to pandemic planning during 1978–2008. We also consulted authors’ personal files and the Internet for records of speeches, national and international conference proceedings, and other unpublished original source documents. In addition to published survey data concerning local and state response planning (*10*,*11*), we sought unpublished data from the Association of State and Territorial Health Officials, the National Association of County and City Health Officials (NACCHO), and the Council of State and Territorial Epidemiologists (CSTE).

## Chronology of US Pandemic Planning

A historical overview of key milestones in US pandemic planning is provided in the Table. In 1977, a federal interagency working group on influenza was formed at the request of the deputy assistant secretary for health in the Department of Health, Education, and Welfare, partly in recognition of the need for greater cooperation across government “silos.” The interagency group included representatives from the Center for Disease Control (CDC; renamed Centers for Disease Control and Prevention in 1992), the National Institutes of Health, the Food and Drug Administration (FDA), and the Department of Defense. Under CDC leadership, the work group drafted the first US pandemic plan, which was released in 1978 and included recommendations for annual influenza immunization of persons at high risk, strengthening of surveillance, expanding research, and establishing a planning and policy mechanism (*12*). 

**Table:** Timeline of selected key events in pandemic planning, United States, 1978–2008

Year	Event	Outcome or follow-up
1978	First US pandemic plan, drafted by Federal Interagency Working Group on Influenza	Planning workgroup and its process assisted in strategy for addressing 1977–78 influenza A(H1N1) outbreak.
1983	Revision of 1978 US pandemic plan	The revised plan laid groundwork for subsequent planning documents.
1988	Institute of Medicine report, The Future of Public Health*	The report recognized the need to improve public health surveillance and response.
1992	Options for the Control of Influenza II meeting, Courchevel, France	The meeting led to formation of the US Federal interagency Group on Influenza Pandemic Preparedness and Emergency Response in 1993.
1997	Publication of elements of the US pandemic preparedness plan in Journal of Infectious Disease	The report updated the action plan, and a further update was published in 2002 in Clinical Infectious Diseases.
1998	CDC emerging infectious disease strategic plan update†	Pandemic influenza was noted as an emerging infection.
1999	Council of State and Territorial Epidemiologists survey data published	Enhanced influenza surveillance was recognized as a cornerstone of pandemic preparedness.
1999	World Health Organization Guidelines for Regional and National Planning	The World Health Organization strongly recommended all countries establish National Pandemic Planning Committees.
2001	Anthrax-related bioterrorism in the United States	The federal response increased state/local preparedness funding.
2003	Severe acute respiratory syndrome outbreaks worldwide	The outbreak led to a globally coordinated response to emerging respiratory pathogens.
2003	Initial detection of human avian influenza A(H5N1) cases in China and Vietnam	The outbreak enhanced attention to pandemic preparedness by Department of Health and Human Services and the US government, accompanied by additional funding.
2005	Department of Health and Human Services pandemic strategic plan	The plan engendered multiple subsequent high-level policy documents and plans from the US government.
2006	Implementation plan for the national strategy for pandemic influenza	This plan led to action steps and a timeline for all pandemic planning pillar areas.
2007	Pandemic influenza vaccine allocation guidance‡	This document preceded the 2009 influenza A(H1N1)pdm09 vaccine recommendations

*http://iom.edu/Reports/1988/The-Future-of-Public-Health.aspx †www.cdc.gov/mmwr/PDF/rr/rr4715.pdf ‡www.flu.gov/images/reports/pi_vaccine_allocation_guidance.pdf

The plan was revised in 1983 to include a new recommendation to develop means to distribute and use influenza antiviral drugs (R.A. Strikas, pers. comm.). Even before completion of the pandemic plan, participants of a 1977 conference on influenza, held by the secretary of the Department of Health, Education, and Welfare, recommended continued federal support for influenza vaccination, particularly to increase vaccination levels of persons at high risk, to improve pandemic preparedness. In addition, CDC implemented a federally funded seasonal influenza immunization program, which purchased 3.4 and 2.4 million vaccine doses for the 1978–79 and 1979–80 influenza seasons, respectively, of which ≈1 million and >1.4 million doses, respectively, were administered. Initial plans were to purchase 8–9 million doses of vaccine. However, budget constraints limited vaccine purchases and ended the program after 1980 (*13*,*14*).

The next major event leading to further US pandemic planning was 1986 legislation creating the National Vaccine Program Office (NVPO), which was given a mandate to coordinate federal vaccine-related activities. At the Options for the Control of Influenza II meeting held in 1992, a consensus report identified the core components of pandemic preparedness: surveillance, vaccines, antiviral drugs, nonmedical/personal hygiene measures, communications, and enhanced annual seasonal influenza vaccination programs (*15*). In 1993, NVPO formed the federal interagency Group on Influenza Pandemic Preparedness and Emergency Response (GrIPPE). The group, which included nonfederal consultants and representatives from CDC, FDA, the National Institutes of Health, and the Department of Defense, drafted a pandemic planning framework that was published in 1997 (*16*) and updated by federal staff in 2002 (*17*). The GrIPPE-initiated planning documents emphasized the need for enhancements to influenza surveillance, vaccine production and distribution, antiviral drugs, influenza research, and emergency preparedness. Perhaps the most consequential outcome of GrIPPE was the creation of a core group of public health experts dedicated to pandemic planning.

Global events helped accelerate interest in pandemic planning. In 1997, Hong Kong recorded the first outbreak of avian influenza A(H5N1) virus infections in humans. Virus was transmitted from infected chickens directly to humans, and 6 of 18 persons with confirmed infection died. In late 1997, >1.5 million chickens were culled throughout Hong Kong as part of successful efforts to stem the outbreak (*18*). This event, combined with the 2003 reemergence of A(H5N1) virus, led to concerns that the next pandemic would be caused by spread of A(H5N1) virus through Asia into Africa and Europe.

In the United States, despite the crucial role of state and local authorities in implementing pandemic plans, a 1995 CSTE survey indicated that <60% of state health departments perceived the need for a state-specific plan (*10*). Through a cooperative agreement between CDC and CSTE, a state and local planning effort was begun in the fall of 1995. The state Project Steering Committee included the GrIPPE co-chairs and representatives from CDC, NVPO, CSTE, and the Association of Public Health Laboratories.

A meeting of >40 state and local health officials convened in September 1996 in Atlanta and identified 4 “pillars” deemed most critical for state and local pandemic preparedness efforts: 1) surveillance, 2) vaccine delivery, 3) communication and coordination, and 4) emergency response. From this meeting and subsequent subgroup meetings dedicated to the 4 pillar areas, critical elements of draft state and local guidelines were developed by January 1997. Four states (Connecticut, Missouri, New Mexico, and New York) and 1 local area (East Windsor Township, New Jersey) were selected by the state Project Steering Committee—primarily on the basis of the identification of a key project leader within each jurisdiction—and funded to pilot test the draft guidelines; 1 additional state, Maine, volunteered to test the draft guidelines without CSTE support. These 5 states conducted pilot tests during February and March 1998 and submitted results to CSTE. Findings were discussed on April 7–8, 1998, at a meeting in Atlanta. The major outcomes from pilot testing were the following recommendations: 1) a fifth pillar area, guidance for use of antiviral drugs, should be added to the guide; 2) the format of the guidelines should be more in concert with the national plan (*18*); and 3) all states should receive the revised guidelines to enable development of state-specific plans (R.A. Strikas, pers. comm.). These 3 issues were discussed at the Association of State and Territorial Health Officials/NACCHO annual meeting in September 1998 and incorporated into the state and local pandemic influenza planning guidelines (R.A. Strikas, pers. comm.), which were then further revised. California, Maryland, Minnesota, and South Carolina were funded through CSTE to develop state plans and submitted their model plans in April 2000.

A national pandemic influenza steering committee was subsequently formed; it was comprised of immunization program managers, emergency preparedness personnel, and representatives from CDC, CSTE, NACCHO, and the Association of Public Health Laboratories (*19*). A national steering committee was a logical extension as the planning process moved from a federal to a national effort.

In 2000, federal funding increased the number of states engaged in pandemic plan development. Florida, Indiana, Massachusetts, New Hampshire, and New Jersey were funded to complete plans by March 2001. In January 2001, Kansas, Washington, Nebraska, Connecticut, and New York were funded to develop plans by March 2002 (*11*). Throughout this process, all states received the same nominal level of funding support, which was typically used to convene a statewide stakeholders meeting. Elements critical to the planning process included technical support provided by the national steering committee and the identification of a key public health professional within each state who assumed responsibility for leading and coordinating planning efforts. Arkansas, Arizona, and Oregon concurrently developed plans of their own accord; West Virginia, Tennessee (1999), and Pennsylvania (1999) had already developed plans. Ultimately, funds were sought for every state to develop a plan.

At this early stage in the planning process, the importance of disseminating information to the broader public health community was recognized. On February 25, 1999, and July 13, 2000, CDC presented satellite videoconferences on influenza pandemic preparedness for states and local areas, which were viewed by >7,000 and ≈6,000 participants, respectively. State and local public health staff engaged in development of pandemic plans participated in the broadcasts. At a meeting of state and local planners sponsored by CSTE and CDC in Atlanta on September 12–13, 2000, detailed discussions were held regarding 1) a scenario of how an influenza pandemic might affect states in 2001; 2) how states should enhance surveillance; 3) how vaccination priorities should be determined, and 4) other national and federal pandemic planning issues, such as infection control, patient triage, and antiviral drug usage (R.A. Strikas, pers. comm.).

After the September 11, 2001, terrorist attacks on the United States, public health preparedness emerged as a priority of the federal government. In 2001, bioterrorism emergency funding support was provided to all states to assist in the nation’s response to the anthrax attacks. The 2003 reemergence of avian influenza A(H5N1) infections in humans fundamentally altered the scale of pandemic preparedness. As the A(H5N1) virus spread to more countries in East and Southeast Asia during 2004–2005, concern grew among senior policymakers and public health experts that the world was on the verge of an influenza pandemic. A(H5N1) infection in humans primarily resulted from exposure to ill poultry and had a case–fatality rate of ≈60%. Substantial federal funding was provided for federal-level planning, procurement of countermeasures (e.g., vaccines and antiviral drugs), development of countermeasures, and state and local pandemic preparedness efforts (*20*). State health departments eventually received $550 million to prepare for an influenza pandemic. Additional high-level policy engagement by the US federal government included the National Strategy for Pandemic Influenza, which was announced in November 2005 (*21*), and the White House’s National Implementation Plan, which was published in May 2006 and addressed federal planning and response strategies: international transport and border control; protection of human and animal health; and security and continuity of operations issues (*22*).

In 2006, the Biomedical Advanced Research and Development Authority (BARDA) was established within the Department of Health and Human Services in response to the growing need for a centralized effort to coordinate research, development, and procurement of countermeasures against potential natural or intentional public health emergencies (*23*). BARDA preparations for a possible A(H5N1) pandemic included development of a stockpile of influenza vaccines produced by using strains circulating in poultry and wild birds in Asia (*24*). In addition, the US government began to purchase influenza antiviral medications for the Strategic National Stockpile sufficient to treat 25% of the US population. Additional investments were initiated to procure ventilators and personal protective equipment, such as respirators.

The US government also initiated an advanced development agenda for vaccines, therapeutics, and diagnostics. BARDA co-invested with industry to modernize vaccine production methods, with the 5-year aim of creating the capacity to produce sufficient vaccine to protect the entire US population within 6 months of the onset of an influenza pandemic (*22*). The US government invested in modernizing diagnostic technologies for public health laboratories. In September 2008, FDA approved specific PCR tests for a panel of influenza diagnostics to be used in CDC reference laboratories in the United States and Department of Defense laboratories around the world. This diagnostic test panel will detect and identify A(H5N1) infections and distinguish novel influenza virus infection from infection with seasonal A, B, and A(H1) and A(H3) influenza viruses. BARDA and CDC awarded contracts in November 2006 for development and evaluation of clinical point-of-care rapid diagnostics to identify seasonal influenza viruses and A(H5N1) viruses (*25*).

Beginning with its first published pandemic plan in 1999 (*26*), the World Health Organization globally promoted pandemic planning among member states, with continued planning efforts thereafter (*27*). The International Partnership on Avian and Pandemic Influenza was formed to coordinate support for developing countries’ efforts to control the spread of A(H5N1) virus and to prepare for an influenza pandemic. This international body convened a series of meetings beginning in January 2006; these efforts generated hundreds of millions of dollars in pledges to support global pandemic preparedness and promoted a level of visibility and readiness that would not otherwise have been possible. In addition to direct financial assistance, the US government provided technical assistance to help countries develop capacities for rapid response, laboratory diagnosis, and surveillance.

The federal government recognized that the foundation for domestic pandemic response rests with state and local governments; thus, the 2005 Department of Health and Human Services strategy and the White House strategy and implementation plan called for major efforts in planning, exercising, and refining state and local preparedness. The 2006 Pandemic and All-Hazards Preparedness Act called for a review of comprehensive state pandemic preparedness plans. The federal government reviewed and scored the plans and released the results to the public in January 2009 (*28*); preparedness levels varied across states and across the domains that were scored. In 2008, as part of its local health profile survey, NACCHO queried local health departments about emergency preparedness and planning activities they had undertaken during the past year (*29*): 89% of 2,332 responding health departments said they had developed or updated pandemic influenza preparedness plans, and 86% said they had participated in tabletop drills or exercises. In addition, 76% had updated their written response plan on the basis of a postexercise after-action report, 72% had participated in a functional drill, and 49% had participated in a full-scale drill or exercise. A total of 68% of local health departments had reviewed existing state legal authorities for isolation and quarantine, and 46% had assessed the emergency preparedness competencies of staff. Only 1% of local health departments did none of the above.

## Evolution of the Pillars of Pandemic Preparedness

The 4 pandemic planning pillars—surveillance, vaccine and antiviral drug delivery, emergency response, and communication—are a solid foundation for pandemic preparation. Although state pandemic plans may have different structures, reliance on these pillars has remained more or less constant across jurisdictions and over time. The major contemporary developments in these core areas are summarized below.

Surveillance, including rapid detection of human infection with novel influenza viruses, remains a cornerstone of pandemic response. This need has been recognized since the early stage of state- and local-based planning (*10*). Improvements in diagnostic technology have enabled confirmation of infection with novel influenza viruses within hours rather than weeks. Human infection with a novel influenza virus became a nationally notifiable disease in 2007, and since then, an increased number of infections have been detected (*30*). Virologic surveillance is also used to determine which seasonal viruses are circulating and thus provides information for seasonal vaccine strain selection. Systems to measure the effect of seasonal influenza (i.e., pediatric deaths, hospitalizations, and syndromic surveillance) have also been enhanced. These systems have been further adapted to measure the effect of pandemic influenza (*31*). The need to maintain ongoing surveillance for novel influenza viruses (e.g., viruses of swine or avian origin) in humans and animals exemplifies the One Health concept (*32*).

In recognition that vaccine might be in short supply during the early phase of a pandemic, federal vaccine allocation guidelines were published in 2008 (*33*). These guidelines laid the groundwork for the pandemic vaccine priority-group recommendations put forth during the 2009 A(H1N1)pdm09 pandemic (*34*). Antiviral medications are critical to a pandemic response, particularly in the interval between recognition of the pandemic and the availability of vaccine. Plans for using these countermeasures have stressed the need for early treatment of affected persons and assumed that the drugs would be scarce.

It was recognized at the 1996 CSTE meeting that close coordination between emergency response staff and public health authorities is needed to develop and implement effective state and local influenza response plans. This recognition has strengthened over time. Although, states were initially not allowed to use bioterrorism funds awarded in 2001 to support pandemic planning, key emergency management concepts, including the all-hazards approach and unified incident command, were eventually integrated into planning efforts (*35*).

Communication, more than ever, is a fundamental component of any response effort. Timely, transparent, and proactive communication is critical, paticularly in the early stages of a confirmed or suspected outbreak, when factual information is limited and the public demand for information and guidance is high. Continuous media coverage and the evolving role of social media (*36*) must be used to enhance communication to and from the public, particularly concerning new or evolving recommendations for disease control.

## Conclusions

Pandemic planning since 2005 had a direct and obvious effect on the response to the 2009 influenza A(H1N1)pdm09 pandemic; however, pandemic preparedness has been a feature of public health since the late 1970s. Coordinated state and federal planning processes have been a consistent feature of that planning. The pillars of pandemic planning response have remained conceptually constant: surveillance; vaccination and delivery of other medical countermeasures; emergency response coordination; and communications.

Although the 2009 A(H1N1)pdm09 pandemic spread globally within a matter of weeks, a 1918-like pandemic did not materialize. Nonetheless, this most recent pandemic resulted in ≈12,500 deaths in the United States, ≈90% of which occurred in persons <65 years of age (*37*). In the wake of this pandemic, the challenge in preparedness is to sustain the interest of private and public sectors in planning for a large-scale outbreak that may have a much more severe effect at a time that cannot be predicted.

Recent assessments of state level epidemiology capacity revealed potentially critical gaps in personnel and training needed for a rapid response to an epidemic (*38*). There will be a need for continued commitments to support state, local, and national planning for the next infectious disease emergency. A comprehensive, coordinated, and effective response cannot be built at the time of a crisis. For future planning and response efforts, sufficient resources are required to sustain the public health response infrastructure developed during the past decade.

An effective response to a pandemic requires at least 4 distinct elements. First, material resources, such as vaccines, antiviral drugs, and personal protective equipment are essential. Second, a commitment to planning, exercising, and refining plans is necessary. Third, a sufficiently large and robustly trained workforce is the basis of any response. Fourth, a commitment to improvement is crucial. This concept extends from continuously improving plans and training to ensuring that scientific advances are incorporated into procurement and planning. One of the main lessons from the history of influenza is to expect the unexpected. Plans and training should be flexible and designed to respond to various levels of disease severity or newly identified pathogens. Benefits from pandemic preparedness will enhance our collective public health response to the next infectious disease crisis.
